# What is the optimal classical style sub-technique during uphill roller skiing in elite male cross-country skiers?

**DOI:** 10.1007/s00421-023-05261-w

**Published:** 2023-07-03

**Authors:** Magne Lund-Hansen, Øyvind Gløersen, Bjarne Rud, Thomas Losnegard

**Affiliations:** 1 Department of Physical Performance, The Norwegian School of Sport Sciences, Ullevål Stadion, Post Box 4014, 0806 Oslo, Norway; 2 Smart Sensors and Microsystems, SINTEF Digital, Oslo, Norway

**Keywords:** Cross-country skiing, Double poling, Kinematics, Kinetics, Maximal oxygen uptake

## Abstract

**Purpose:**

To compare performance, physiological and biomechanical responses between double poling (DP) and diagonal stride (DIA) during treadmill roller skiing in elite male cross-country skiers.

**Method:**

Twelve skiers (*V*O_2peak_ DIA_up_; 74.7 ± 3.7 ml kg^−1^ min^−1^) performed two DP conditions at 1° (DP_flat_) and 8° (DP_up_) incline, and one DIA condition, 8° (DIA_up_). Submaximal gross efficiency (GE) and maximal 3.5 min time-trial (TT) performance, including measurements of *V*O_2peak_ and maximal accumulated O_2_-deficit (MAOD), were determined. Temporal patterns and kinematics were assessed using 2D video, while pole kinetics were obtained from pole force.

**Results:**

DIA_up_ induced (mean, [95% confidence interval]) 13% [4, 22] better 3.5-min TT performance, 7%, [5, 10]) higher *V*O_2peak_ and 3% points [1, 5] higher GE compared to DP_up_ (all *P* < 0.05). DP_up_ induced 120% higher MAOD compared to DP_flat_, while no significant differences were observed for *V*O_2peak_ or GE between DP_flat_ and DP_up_. There was a large correlation between performance and GE in DP and a large correlation between performance and *V*O_2peak_ for DIA_up_ (all *r* = 0.7–0.8, *P* < 0.05). No correlations were found between performance and VO_2peak_ for any of the DP conditions, nor between performance and GE for DIA_up_ (*r* = 0.0–0.2, *P* > 0.1).

**Conclusion:**

At 8º uphill roller skiing, DIA_up_ induce higher *V*O_2peak_, GE, and superior time-trial performance than DP_up_ in elite male skiers. There was no difference between *V*O_2peak_ or GE between DP_flat_ and DP_up_. A large correlation was observed between DIA_up_ performance and DIA_up_ *V*O_2peak_, while DP performance was best correlated to submaximal GE.

## Introduction

Classical cross-country skiing consists of different subtechniques. The two main subtechniques used in competitive skiing are double poling (DP) and diagonal stride (DIA). Traditionally, DP has been used at higher speeds and in flatter terrain, while DIA has been used at lower speeds and in steeper terrain (Pellegrini et al. 2013). However, due to improvements in equipment and race-track preparation, there has been a substantial increase in race speed over the last decades along with elite skiers using DP exclusively during races (Losnegard 2019). Consequently, to “protect classical technique and all its aspects” rules decided by the International skiing federation restricted use of DP in specified sections of uphills, referred to as “technique zones”, where only use of diagonal stride or herringbone techniques are permitted. Despite these rules, skiers may still race without grip wax, with the use of a special uphill technique—like herringbone, with the main requirement being that the skies are not allowed to glide over the snow, whenever DP is forbidden. Skiers can therefore still take advantage of lower ski-snow friction in the flat and downhill sections, and thereby enhance overall performance.

During DP, all propulsive forces are transferred through the poles, resulting in a significant contribution from the upper body muscles (Bojsen-Møller et al. 2010; Danielsen et al. 2019; Holmberg et al. 2005; Lindinger et al. 2009). In addition, considerable work is done by the muscles in the lower limbs to raise and extend the body to an upright position during the repositioning phase (Bojsen-Møller et al. 2010; Danielsen et al. 2019; Holmberg et al. 2005). This contribution from the lower limbs is crucial as it enables greater external power and speed during the poling phase (Danielsen et al. 2019; Holmberg et al. 2006). The ratio between upper versus lower body contribution changes with different speeds and inclines due to differences in the vertical displacement of the center of mass (COM), poling time, peak poling force, and joint kinematics within DP (Danielsen et al. 2019; Rud et al. 2014). Thus, within DP, elite skiers benefit from using several “gears”, with various combinations of cycle rates and cycle lengths at a given incline and/or speed, to optimize propulsion (Dahl et al. 2017; Danielsen et al. 2021). Although recent studies have compared DP and DIA in uphill skiing (Andersson et al. 2021; Sagelv et al. 2018; Stoggl et al. 2019; Stoggl and Holmberg 2016), our understanding of technique selection from a combined performance, and physiological and biomechanical perspective in elite skiers remains limited.

In DIA, skiers exert force through the skis and the poles in a similar pattern to running with poles (Kehler et al. 2014). This is the sub-technique that elicits the highest *V*O_2peak_ (Losnegard et al. 2014), with an average of 12% (range 5–18%) higher *V*O_2peak_ being achieved in DIA compared to DP, independent of performance level (Losnegard 2019). Several factors appear to contribute to this difference, including lower oxidative capacity of the arm muscles (Calbet et al. 2005; Rud et al. 2014), limited time to produce power due to the short poling phase, less muscle mass involvement, and a domination of the arm vs. leg muscles (Losnegard 2019). However, the three latter suggestions may be altered by increasing the steepness of the terrain, thereby leading to a potential greater contribution of the legs in DP due to more vertical displacement of COM and longer poling duration (Danielsen et al. 2019; Stoggl and Holmberg 2016). It could also potentially increase the use of muscle mass and thereby the ability to sustain a higher workload through greater maximal accumulated oxygen deficit (MAOD). When comparing physiological differences between DP and DIA, it is therefore important to take into consideration variations in incline and the resulting movement pattern used in DP (Danielsen et al. 2019; Stoggl and Holmberg 2016).

Performance in cross-country skiing is highly related to peak aerobic power (*V*O_2peak_) and the energy cost of locomotion. Moreover, anaerobic capacity, tested as MAOD, seems an important factor, at least for sprint skiers (Losnegard et al. 2012). The relative contribution of these different performance determinants appears to vary, not only between events (sprint < 1.8 km versus distance > 10 km), but also depending on which sub-technique is employed (Losnegard 2019; Skattebo et al. 2019). Skattebo et al. (2019) found that World Class long-distance skiers (> 40 km) who were highly specialized in DP had a lower energy cost when utilizing DP at the same velocity compared to World class distance skiers, despite no major biomechanical differences. Similar results were found in Torvik et al. (2022), who compared World Class long-distance skiers with a group of distance skiers. They found a difference in the ratio between *V*O_2peak_ in DP and running between the groups, but there was also a difference in the performance level in the two groups. In general, the difference between *V*O_2peak_ in DP and DIA (or running) is therefore found to be 4–18%, independent of performance level and does not appear to decrease as a result of specialized DP training (Losnegard 2019; Torvik et al. 2022). Therefore, it could be proposed that DP performance is less related to maximal oxygen uptake than in other skiing techniques. However, to date, this has not been thoroughly investigated in elite skiers.

The aims of the current study were therefore to investigate: (1) performance and physiological differences between DP and DIA during uphill skiing on a treadmill in elite male skiers; (2) physiological and biomechanical differences between two DP conditions (flat and uphill); (3) the relationship between performance and physiological determinants in DP versus DIA.

## Materials and methods

### Subjects

Twelve elite male skiers (mean ± SD: age 23 ± 5 years; height, 180 ± 5 cm; body mass, 73 ± 6 kg) participated in the study. To be included, skiers had to meet at least one of the following criteria: (1) participated in the Norwegian senior national championship (2) top 30 in their age group for juniors in Norway, or (3) top 30 in one of the major races in the long-distance skiing Visma Ski Classics. Six of the skiers were specialized long-distance (e.g., competing in longer, 20–95 k, races with DP exclusively) skiers and six were traditional distance (e.g., competing in the Olympic distances) skiers. The local ethical committee of the Norwegian School of Sport Science approved the study. The project was conducted according to the Declaration of Helsinki and all participants gave written informed consent.

### Experimental overview

All tests were completed on a 1.0 × 2.7 m (Rodby, Södertalje, Sweden) roller ski treadmill. Skiers were familiarized to the treadmill before conducting two test sessions separated by 5–7 days. During the main protocol, participants completed three submaximal workloads in DP at 8° (named uphill; DP_up_), in DP at 1° (named flat; DP_flat_) and DIA at 8° (named DIA_up_) to determine gross efficiency (GE). Speed was set individually to target a Borg scale rating (rate of perceived exertion; RPE; 6–20) (Borg 1982) of 11–12, 13–14 and 15–16 for DP_up,_ DP_flat_, and DIA_up_, respectively (Losnegard et al. 2021) based on the familiarization session. Maximal 3.5 min time-trial (TT) tests were conducted at the same inclines to determine *V*O_2peak_, maximal accumulated oxygen deficit (MAOD) and performance in the different conditions.

### Test protocol

All subjects used the same pair of roller skis (IDT solutions AS, Lena, Norge) with wheel type 3 and an NNN-binding system (Rottefella, Lier, Norway) and Swix Triac 1.0 poles (Swix, Lillehammer, Norway) with a roller skiing tip. The roller skis had a coefficient of rolling resistance of 0.027, which did not change during the testing period. Friction was measured using a towing test, previously described by Hoffman et al. (1990).

For all three sessions, skiers completed a low-intensity 10 min warm-up on the treadmill at 2° incline and 3.5 m s^−1^ at a heart rate (HR) of ⁓ 60–70% of maximal HR. On the first day, subjects completed three submaximal workloads in DP_flat_ and DP_up,_ respectively. For each workload, the speed was increased equal to ~ 20 W and had a duration of 5 min load and 2 min recovery. Independent of speed on the first submaximal workload, the skiers had the same increase in speed between each workload. The VO_2_ for calculation of GE was determined as the average from 3 to 5 min. HR was averaged over the same period. RPE and La^−^ were recorded after each workload. After the last submaximal workload, participants were given a 10 min recovery, before they performed a 3.5 min maximal TT at an initial speed of 2.39 m s^−1^ for the first 30 s, after which speed was self-selected. The velocity of the treadmill was increased when the skis were in front of a laser beam across the front section of the treadmill and was decreased if the skis fell behind a second laser beam further back on the treadmill. Oxygen uptake was measured continuously throughout the test, and the highest average measure over 30 s was defined as *V*O_2peak_. Highest heart rate (HR_peak_) and La^−^ were obtained after the test.

On the second day, subjects completed three submaximal DIA_up_ workloads, similar to the protocol completed on DP_up_. After a 10 min recovery, subjects completed the DP_flat_ and DP_up,_ maximal TT separated by a 20 min recovery. *V*O_2_, HR, and La^−^ were recorded as described for the first day. Accumulated oxygen demand was estimated by extrapolation of the individual linear relationship between the work rate (W) and steady-state O_2_-cost from the submaximal loads. MAOD was calculated as the difference between accumulated oxygen demand and accumulated oxygen consumption during the entire TT (Losnegard et al. (2012).

### Apparatus

Oxygen consumption was measured using an automatic ergospirometry system with mixing chamber (Oxycon Pro, Jaeger GmbH, Hoechberg, Germany), as evaluated by Foss and Hallen (2005). Capillary blood for measurement of La^−^ was taken from the finger and analyzed using Biosen C-line (EKF Diagnostics, Cardiff, England). Calibration was performed automatically every hour with a 12 mmol/L solution (Biosen Multi standard solution 12 mmol/L, EKF Diagnostic, Cardiff, England). Subjects used their own heart rate monitors.

For biomechanical analyses, markers (white sports tape with black circular marks) were attached at the following anatomical landmark: Acromion, lateral epicondyle of the elbow, ulnocarpal joint of the hand, trochanter major, lateral epicondyle of the knee, lateral malleolus, and over the fifth metatarsal on the ski boot, before the submaximal tests (Carlsen et al. 2018). Sagittal plane kinematics were recorded based on video recordings from the right side for the first 30 s of each of the DP workloads. 2D video analysis in the sagittal plane has been shown to have good reliability compared to 3D motion capture for basic exercises (Gribble et al. 2005; Norris and Olson 2011). None of the markers were moved between the different DP conditions. Video was collected using an iPad Pro (Apple, Cupertino, California, USA) with a frame rate of 120 Hz. The iPad was mounted on a tripod positioned perpendicular to the skiing direction and the distance between the camera and skiers was 3 m.

Resultant pole force was collected via one final work interval following the main testing protocol. Six of the skiers performed 60 s at the highest submaximal workload in both DP conditions. Pole-force was measured with a custom pole handle (Polar Electro OY, Kempele, Finland). The handles had a sampling frequency of 260 Hz, which was downsampled to 100 Hz before the analyzing. The data shown are an average of six poling cycles.

### Data analysis

Kinematic data for DP_flat_ (6.8 ± 0.6 m s^−1^) and DP_up_ (1.8 ± 0.2 m s^−1^) were analyzed from the highest submaximal workload. Tracker (Open Source Physics, USA) was used to digitize anatomical landmarks to calculate sagittal plane ankle, elbow, shoulder, knee, and hip joint angles. The digitized trajectories were low-pass-filtered (second-order bidirectional Butterworth filter, cutoff 12 Hz), resampled to 101 data points for each individual cycle, and are presented as the average of five consecutive cycles. The low-pass filter cutoff was based on a residual analysis. Vertical COM position (zCOM) was determined using relative segment weights from De Leva (1996), and measured with respect to a fixed point on the treadmill band, to be consistent with overground locomotion. This was done, so that zCOM reflects changes in gravitational potential energy with respect to the treadmill band the skier is moving over and was determined using trigonometry based on the treadmills speed and incline. All joint angles and zCOM were collected at the same external workload for DP_flat_ and DP_up_. The pole force measurements were not filtered. Pole contact was defined as pole force > 15 N.

External power was calculated as explained in Sandbakk et al. (2010) as the rate of change in gravitational potential energy plus the rate of energy lost to rolling resistance.

GE at submaximal workloads was calculated as the ratio between external power and the metabolic rate (converted to watts), and expressed as a percentage (Losnegard et al. 2014). Aerobic metabolic rate was determined based on *V*O_2_ and the corresponding RER-value together with a standard table for conversion (Péronnet and Massicotte 1991). MAOD was calculated as previously done by Losnegard et al. (2012), by subtracting the average V̇O_2_ from the average O_2_ demand of the 3.5 min TT.

### Statistical analysis

Normality of the data was assessed using the Shapiro–Wilk test (*α* = 0.05). Data are presented as relative values as mean ± 95% confidence interval (CI). A two-way repeated-measures ANOVA was used to assess interaction and main effects of technique and external powers for submaximal workloads (3 × 3 design). In case of significant effects, multiple comparisons with Tukey post hoc correction was used. To test differences between the subtechniques during the maximal test, a one-way ANOVA for repeated measurements with a Tukey post hoc test was used. Differences in kinematic data between DP up and flat were analyzed using Student’s *T *test. Pearson product–moment correlation was used to determine correlation between performance and physiological parameters. The strength of the correlation was assessed based of the following range: < 0.1 negligible correlation, 0.1–0.3 small correlation, 0.3–0.5 moderate correlation, 0.5–0.7, large correlation, 0.7–0.9, very large correlation, and 0.9–1.0 almost perfect correlation (Hopkins 2000).

Statistical calculations were performed using Microsoft Office Excel 2013 (Microsoft, Redmond, USA) and Graph Pad prism 8.2.1 (San Diego, CA, USA). The level of significance was set at α = 0.05.

## Results

### Performance and physiological differences between DIA and DP

**Fig. 1:**
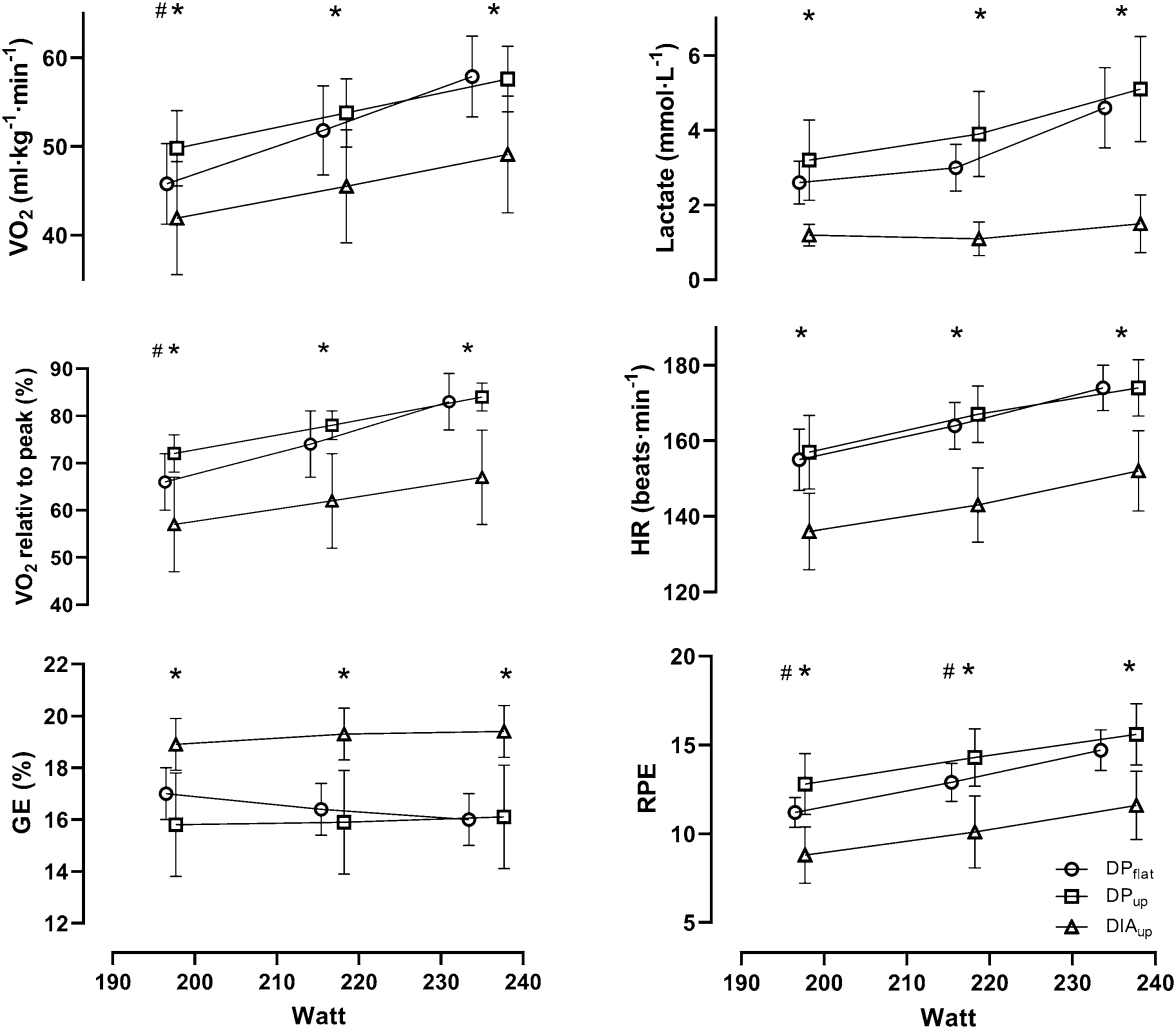
Submaximal O_2_-cost, blood lactate concentration, O_2-_cost relative to *V*O_2peak_, gross efficiency (GE), heart rate, and rate of perceived exertion (RPE) at the different techniques. *Significant difference between DIA and both DP conditions (*P* < 0.05). ^#^Significant difference between DP_flat_ and DP_up_ (*P* < 0.05)

The 3.5 min TT distance was 13% [4, 22] longer for DIA_up_ compared to DP_up_ (610 m vs. 550 m). Peak oxygen uptake was 7% [5, 10] higher during DIA_up_ than both DP_flat_ and DP_up_ (Table 1), while no significant difference was found between DP_flat_ and DP_up_. MAOD was greater during DP_up_ than both DIA_up_ and DP_flat_ (Table 1). We observed an interaction effect between external power and technique at submaximal workloads for La^−^, VO_2_, and GE (all *P* < 0.001), HR (*P* = 0.049) and RPE (*P* = 0.020), as well as a main effect of both on all variables (*P* < 0.001) except for external power on GE (*P* = 0.650). Post hoc tests revealed that La^−^, HR, RPE, and VO_2_ were all lower and GE higher for DIA_up_ than DP_up_ and DP_flat_ (Fig. 1,* P* < 0.001**).** No significant differences between the two DP conditions were observed in La^−^, HR, or GE at any of the workloads. RPE was lower for DP_flat_ than for DP_up_ at the first two workloads (*P* < 0.001), but not at the last (Fig. 1, *P* = 0.080)_._

**Table 1:** Performance and physiological response to a 3.5 min all-out test during treadmill roller skiing

	DP_flat_	DP_up_	DIA_up_	*P* value
External power (W)	255 ± 31	331 ± 43	363 ± 34	< 0.001
*V*O_2peak_ (ml kg^−1^ min^−1^)	69.5 ± 2.4	68.6 ± 2.9	74.7 ± 3.7	< 0.001
Mean O_2_ uptake (ml kg^−1^ min^−1^)	56.2 ± 2.6	55.7 ± 2.3	60.5 ± 2.5	0.002
O_2_ demand (ml kg^−1^ min^−1^)	65.8 ± 3.9	75.4 ± 3.2	70.9 ± 6.4	0.007
MAOD (ml kg^−1^)	30 ± 12	68 ± 10	36 ± 16	< 0.001
[La^−^] (mmol L^−1^)	9.6 ± 1.6	10.9 ± 1.9^a^	10.1 ± 1.7	0.038
VE_peak_ (L min^−1^)	185 ± 18	187 ± 23	194 ± 19	0.022
HR_peak_ (beats min^−1^)	186 ± 6	187 ± 7	189 ± 7	0.017
RER	1.10 ± 0.13	1.12 ± 0.06	1.12 ± 0.04	0.332

Data are mean ± SD, *N* = 12, except ƩO_2_-deficit and heart rate (HR) (*N* = 9)

*DP*_*flat*_ double poling at 1°, *DP*_*up*_ double poling at 8°, *DIA*_*up*_ diagonal stride at 8°, *[La*^*−*^*]* blood lactate concentration, *VE* ventilation, *HR* heart rate, *RER* respiratory exchange ratio, *MAOD* maximal accumulated oxygen uptake

Level of significant (*P*) from one-way ANOVA for repeated measurements

### Kinematic and kinetic comparison between DP_flat_ and DP_up_

**Fig. 2:**
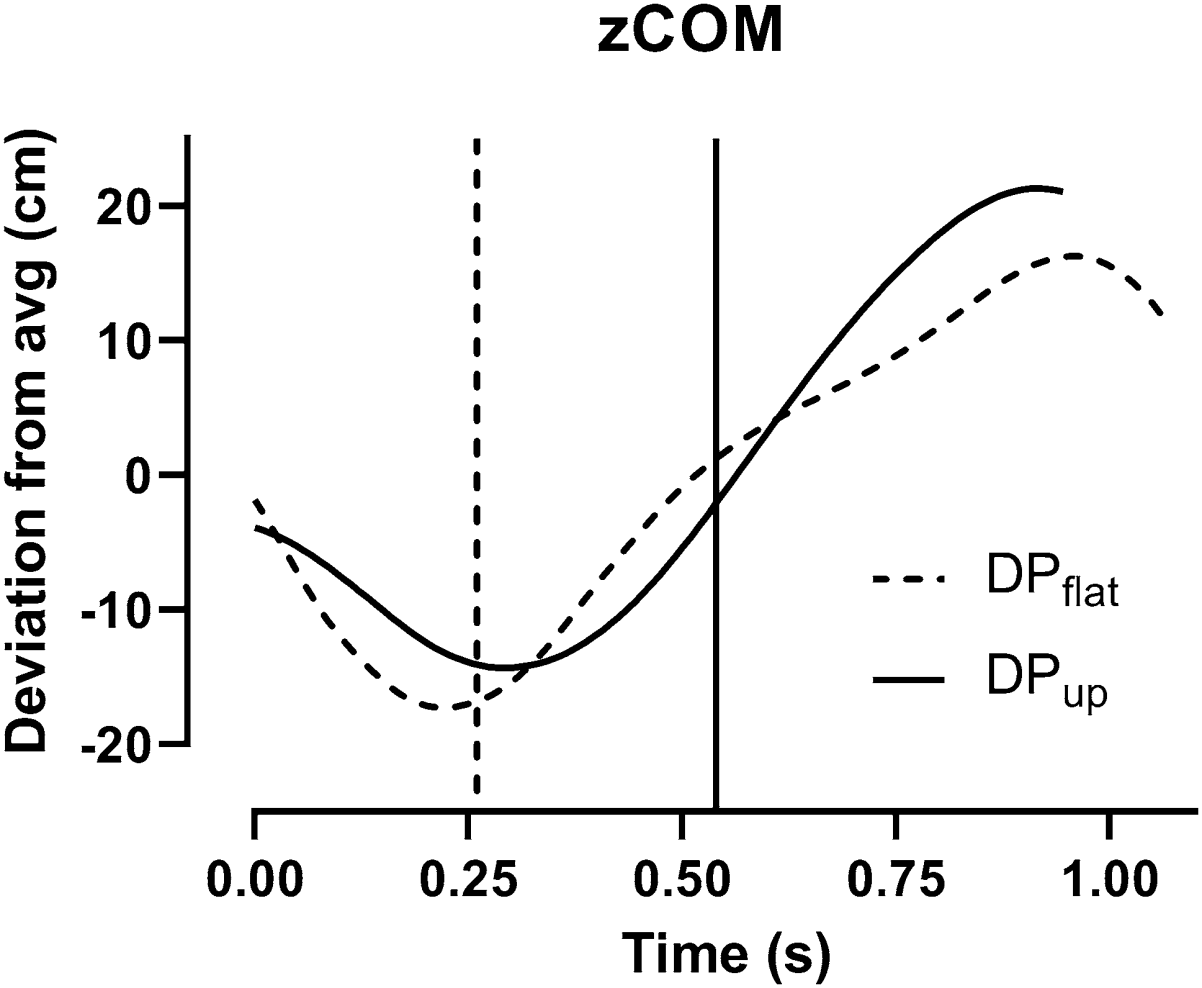
Vertical movement of COM from the last submaximal workloads for double poling (DP). The lines mark the point where the pole tip leaves the treadmill. zCOM is presented as the difference from the average placement of the COM. Movement along the treadmill incline is brought into the calculation. All data are presented as average (*n* = 11)

**Fig. 3:**
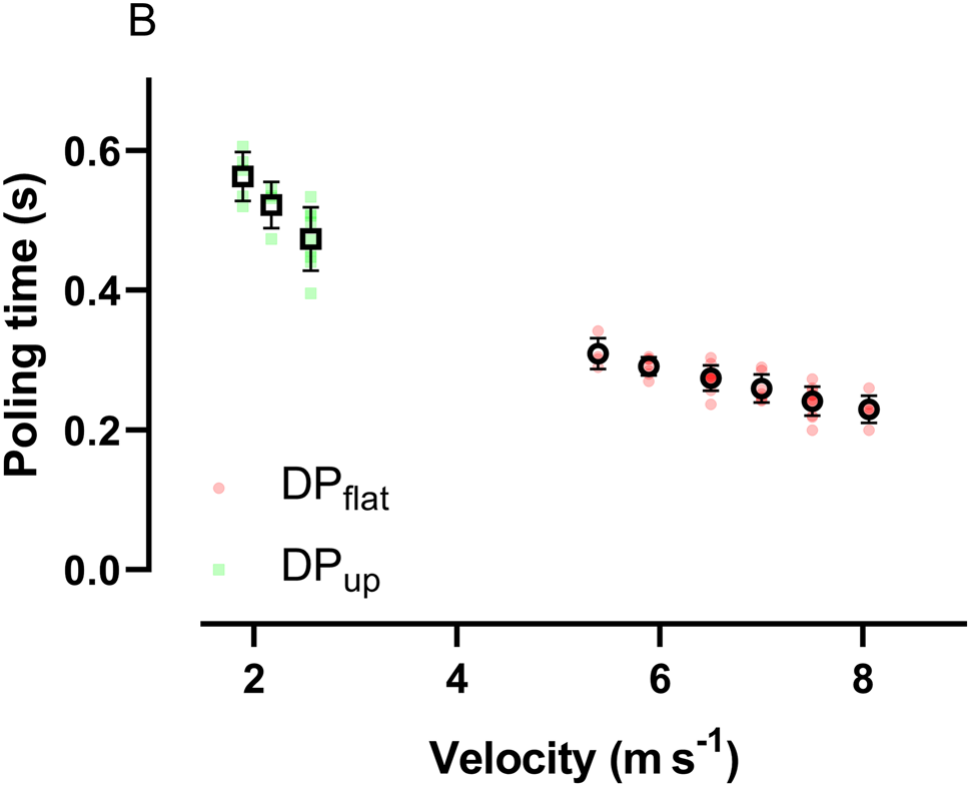
Poling time during different workloads for DP_up_ and DP_flat_. Poling time is calculated based on the time the pole tip is in contact with the treadmill

**Fig. 4:**
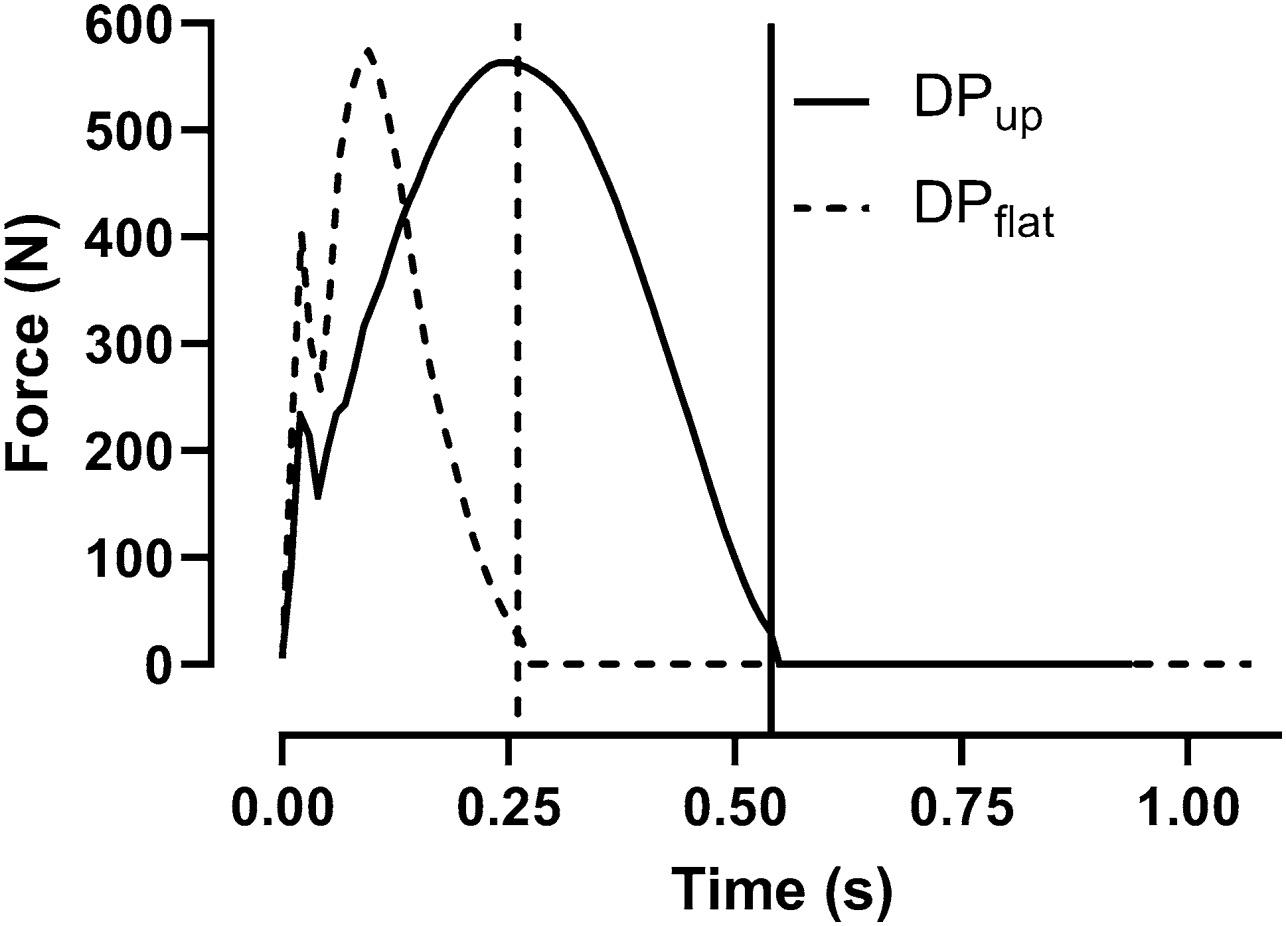
Resultant pole force during the poling phase. The force data from the poles were collected at the same speed as the kinematic data. Force is presented as average force from two poles (*n* = 6)

The kinematic data are presented in Table 2 and Fig. 2. DP_flat_ had a longer cycle time and reposition time, but shorter absolute and relative poling time than DP_up_. The relationship between speed and poling time is shown in Fig. 3. DP_flat_ induced a greater extension during the reposition phase which resulted in a greater lowering of zCOM before pole plant compared to DP_up_ (Fig. 2). DP_flat_ also induced a greater lowering of zCOM from pole plant to cycle minimum, but a less zCOM increase from cycle minimum to maximum, compared to DP_up_. Ankle and knee angle ROM was greater for DP_up_ compared to DP_flat_, while hip, shoulder, and elbow angle ROM was smaller for DP_up_ compared to DP_flat_ (Table 2). The resultant pole force relative to cycle time during DP_up_ and DP_flat_ is presented in Fig. 4**.** There was no significant difference in peak force (295 ± 48 N vs. 285 ± 35 N, *P* = 0.3), but DP_flat_ had a higher impact pole force (210 N ± 29 vs. 117 N ± 14, *P* < 0.001) than DP_up_. Peak force was reached earlier during the poling phase with DP_flat_ than DP_up_ (37 ± 2. % vs. 47 ± 5%). The resultant force impulse was higher for DP_up_ than for DP_flat_ (193 ± 19 N∙s vs. 79 ± 5 N∙s, *P* < 0.001).

**Table 2:** Speed, external power, and kinematic data from double poling (DP) flat (1°) and uphill (up) (8°) (*n* = 11)

	DP_flat_	DP_up_	*P *value
Speed m s^−1^	6.8 ± 0.6	1.8 ± 0.2	< 0.001
Power (W)	229 ± 37	229 ± 39	0.335
Poling time (s)	0.26 ± 0.02	0.54 ± 0.04	< 0.001
Reposition time (s)	0.81 ± 0.08	0.41 ± 0.04	< 0.001
Cycle time (s)	1.07 ± 0.10	0.94 ± 0.06	< 0.001
% Poling time	25 ± 1	57 ± 2	< 0.001
ROM ankle (°)	14 ± 6	20 ± 8	0.002
ROM knee (°)	33 ± 12	37 ± 14	0.010
ROM hip (°)	84 ± 30	63 ± 23	< 0.001
ROM elbow (°)	102 ± 36	49 ± 19	< 0.001
ROM shoulder (°)	98 ± 28	53 ± 22	< 0.001

### Determinants of DIA and DP performance

**Fig. 5:**
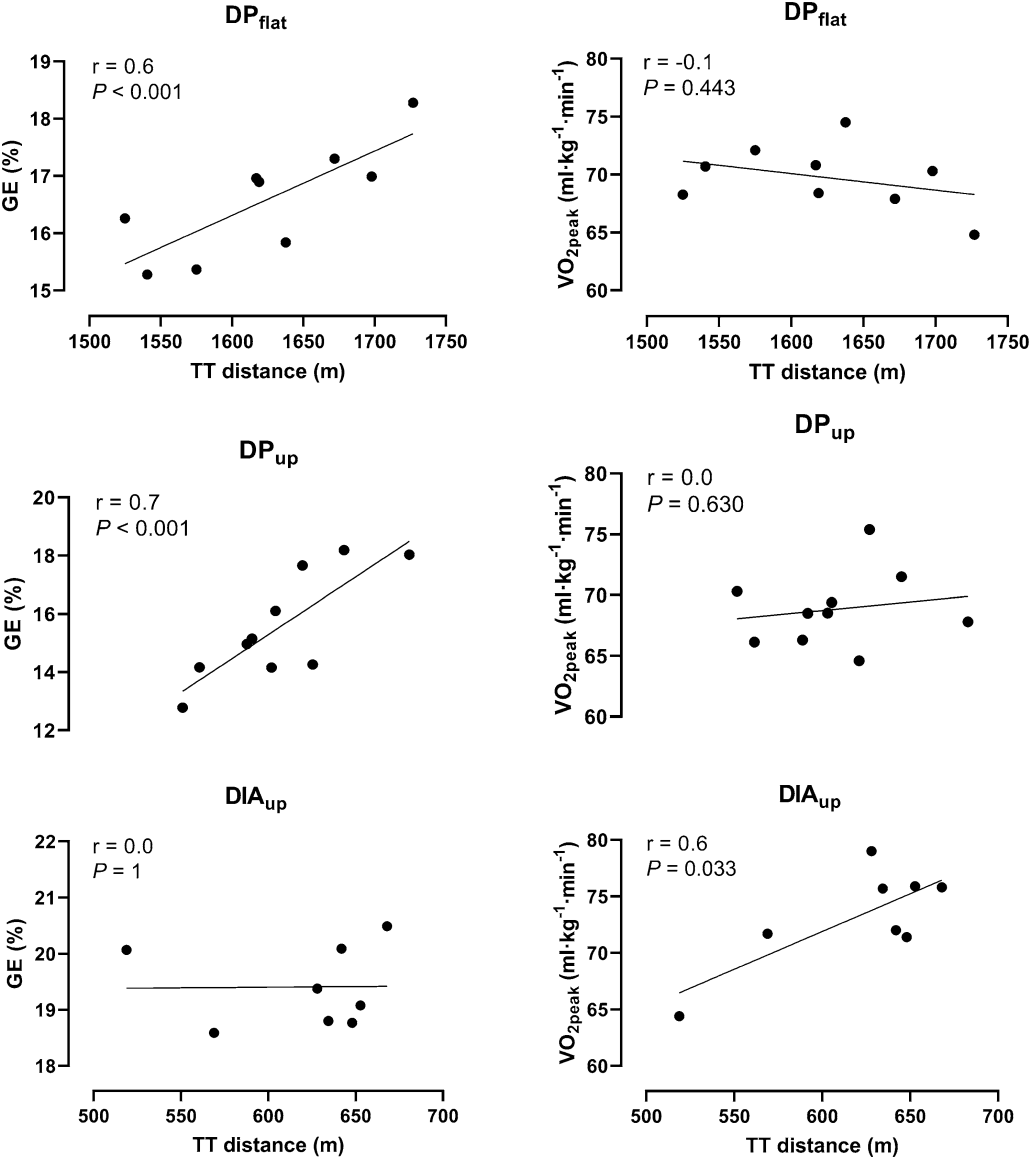
Correlation between performance and gross efficiency (GE) and *V*O_2peak_ for DP_flat_, DP_up_ and DIA_up_. GE is an average of the three submaximal workloads. *Significant correlation (*P* < 0.05)

Correlations between 3.5 min TT performance and physiological determinants of performance from the different techniques are presented in Fig. 5. There was a large correlation between performance in the TT and GE at the last of the three submaximal workloads in DP_flat_ (*r* = 0.6, *P* < 0.001) and DP_up_ (*r* = 0.7, *P* < 0.001). We found no correlation between performance and *V*O_2peak_ in DP_flat_ (*r* = − 0.1, *P* = 0.443) and DP_up_ (*r* = 0.0. *P* = 0.630). There was no correlation between performance in the TT and GE at the last of the three submaximal workloads in DIA_up_ (*r* = 0.0, *P* = 1). There was a large correlation between TT performance and *V*O_2peak_ for DIA_up_ (*r* = 0.6, *P* = 0.033).

## Discussion

This study investigated performance, physiological and biomechanical differences between the two classical style techniques DIA and DP in elite male skiers. The main findings were that; (1) DIA resulted in better performance than DP in the uphill test and was accompanied by a higher submaximal GE and a higher *V*O_2peak_. (2) No differences were seen in VO_2peak_ between DP flat and uphill, while longer poling time and higher maximal accumulated oxygen deficit were evident in DP uphill versus DP flat. (3) There was a strong correlation between performance and VO_2peak_ in DIA_up_ and a strong correlation between performance and submaximal GE in DP.

Higher *V*O_2peak_ and higher GE in DIA_up_ versus DP were accompanied by a significantly better performance in the 3.5 min time-trial test. The present study, therefore, indicates that the use of DIA is superior compared to DP during uphill roller skiing, which is in agreement with the previous studies (Dahl et al. 2017; Hoffman et al. 1994; Pellegrini et al. 2013; Sagelv et al. 2018). However, these results may not be directly transferable to skiing on snow. Despite the clear physiological and biomechanical disadvantages for DP versus DIA in uphill treadmill skiing observed in the present study, elite skiers frequently use DP on snow in uphill terrain (Stoggl et al. (2019). The reason for this might be related to improved glide in the absence of grip wax, while DIA is dependent of grip wax on the skis. Roller skis provide “perfect grip” (locked rear wheel when kicking backwards) without any compromise on rolling abilities. From a practical viewpoint, this illustrates the importance of combining aspects of physiology, biomechanics, and equipment to better facilitate more specific training and increase performance.

Despite increasing focus on specialized DP training the last decades, the physiological differences between DP and DIA observed in the present study are similar to the previous reports (Andersson et al. 2021; Dahl et al. 2017; Sagelv et al. 2018). We found a difference of 7% in the *V*O_2peak_ DP/DIA ratio, which is within the 4–18% range documented previously (Losnegard 2019; Torvik et al. 2022). Among our subjects, half (6) were distance skiers (DS), while the other half were specialized long-distance skiers (LDS) whose training primarily focuses on increasing DP performance (Torvik et al. 2021). However, we found no difference in *V*O_2peak_ between these two subgroups of skiers, which is in line with Skattebo et al. (2019). On the other hand, others have reported differences in *V*O_2peak_ between LDS and DS (Torvik et al. 2022). However, in Torvik et al. (2022), both DS and LDS had lower *V*O_2peak_ values compared to the present and previous (Skattebo et al. (2019). Although the LDS appear as elite in Torvik et al. (2022) that did not report the performance level for DS and there were few subjects for each group, with 5 LDS and 7 DS. Overall, performance level between groups with different specialization may differ from study to study, and such differences in findings across studies, therefore, suggest that skiing level is an important determinant when comparing performance parameters between LDS and DS. Overall, these studies suggests that the increased specialization in DP over the last decades might induce other adaptations than only “closing the gap” in the DIA/DP *V*O_2peak_ ratio (Losnegard 2019; Torvik et al. 2022).

Interestingly, we found a large correlation between DIA_up_ performance and *V*O_2peak_, while this was not evident for DP performance and *V*O_2peak_ in either flat or uphill. However, a strong correlation (*r* = 0.7) was found between DP performance and DP submaximal GE in both conditions. This relationship supports the findings of Sagelv et al. (2018), Skattebo et al. (2019), and Torvik et al. (2022) that the energy cost of locomotion is of particular importance for DP performance. Furthermore, the “weak” relation between *V*O_2peak_ and DP performance fits well with the previous findings of blunting of whole-body maximal oxygen uptake, when an increasing part of the external work is performed by the upper body and arms (Calbet et al. 2005, 2004). To sum up, this suggests that “fine tuning” of the DP technique adds more to increased DP performance in elite skiers than improvements of physiological factors important for increasing DP peak oxygen uptake.

We observed no difference in *V*O_2peak_ between DP_up_ and DP_flat_ despite a greater power output in DP_up_ during the performance test (330 W versus 255 W). The greater energetic cost for DP_up_ compared to DP_flat_ is likely reflected by greater MAOD (Table 1). This is in line with the previous results conducted in roller ski skating where a significantly higher MAOD was observed when skiing uphill (8°) versus flat (1°) (Karlsson et al. 2018). The difference in MAOD for flat vs. uphill terrain can be explained by at least two important factors. First, in DP uphill, skiers increase the lower body work (Danielsen et al. 2019) and thereby the activation of the lower body muscles (Rud et al. 2014). Indeed, on flat terrain, DP requires an ability to convert gravitational potential energy from the highest (pre-pole plant) to lowest position into forward movement (Fig. 2). However, increased incline reduces the gravitational energy that can be used during the ground contact phase, since the ground level is higher at the termination of the contact phase than at the beginning. For the same reason, more work against gravity must be done during the reposition phase of uphill double poling. In our results, this is seen by a smaller fall in zCOM position during the poling phase, and a greater increase in zCOM position during the reposition phase, in DP_up_ compared to DP_flat_. The short reposition time during DP_up_ suggests the need to finish the lowering of zCOM earlier and use of the arms to help lift the body back into position before pole plant. Furthermore, the increased joint angle ROM in the lower extremities (knee and ankle) and reduced ROM in the upper body (hip, shoulder, and elbow) in DP_up_ compared to DP_flat_ indicates that the lower body contribution to DP becomes more important as incline increases. This increased muscle activation can have an influence on the MAOD of the movement (Olesen 1992).

Second, DP_up_ is associated with a significantly longer poling time, compared to DP_flat_ (Dahl et al. 2017; Danielsen et al. 2019; Lindinger et al. 2009; Nilsson et al. 2004; Stoggl & Holmberg 2016). The short poling time in DP_flat_ (Fig. 3) is disadvantageous for the force–velocity properties of the muscles, limiting high external power production (Hill 1938). Moreover, in the present study, peak force occurred later during the poling phase for DP_up_ than DP_flat_ (Fig. 4). Together with the 9% higher contribution from the legs and trunk during DP at 12% inclination compared to DP at 5% observed in Danielsen et al. (2019), this may reduce muscle contractile velocity and increase the muscles’ ability to produce force in DP_up_ compared to DP_flat_. These alterations in technique would allow one to perform more work per cycle during DP_up_ relative to DP_flat_.

### Practical applications

The present study provides novel insights regarding the association between physiological (i.e., *V*O_2peak_, O_2_-cost, and MAOD) and biomechanical (i.e., kinematics and kinetics) properties during different subtechniques (DIA and DP) and how this influences performance in classical cross-country skiing. Regardless of the increased usage of DP over DIA in uphill sections of ski racing, DIA seems to be superior to DP on steep uphill sections. This implies that skiing with no grip wax (DP only) is the major reason for skiers choosing DP throughout races, as the increased speed in flatter and downhill sections makes up for eventual losses during steep uphills. Importantly, despite the similarities between roller skiing and on snow skiing, there are notable differences regarding grip and glide. Such differences must be taken into consideration when interpreting these results.

The DIA/DP *V*O_2peak_ ratio between the present and previous studies, together with the correlations between GE/*V*O_2peak_ and performance in DIA and DP in the present study, implies that economy and durability is more important than *V*O_2peak_ for improving DP performance in elite skiers, it is more important to improve work economy or efficiency, that is, technique rather than *V*O_2peak_. Therefore, it is important to consider the limiting factors for performance when utilizing the DP technique compared to other subtechniques. This should be taken into consideration when designing training programs for athletes.

## Conclusion

**At 8º incline**, DIA induces higher *V*O_2peak_ and GE compared to DP in elite male skiers. Moreover, DIA resulted in superior performance compared to DP during uphill roller skiing. Finally, a large correlation was observed between DIA_up_ performance and DIA_up_ VO_2peak_, while performance was best correlated to submaximal GE for both DP conditions.

## Data Availability

The datasets generated during and/or analyzed during the current study are available from the corresponding author on reasonable request.

## Abbreviations

ANOVA: Analysis of variance
COM: Center of mass
DIA_up_: Diagonal stride at 8°
DP_flat_: Double poling at 1°
DP_up_: Double poling at 8°
GE: Gross efficiency
HR: Heart rate
La^−^: Blood lactate concentration
MAOD: Maximal accumulated oxygen deficit
ROM: Range of motion
RPE: Rate of perceived exertion
TT: Time-trial
*V*O_2peak_: Highest oxygen uptake over 30 s
zCOM: Vertical displacement of center of mass
